# Event-driven visual attention for the humanoid robot iCub

**DOI:** 10.3389/fnins.2013.00234

**Published:** 2013-12-13

**Authors:** Francesco Rea, Giorgio Metta, Chiara Bartolozzi

**Affiliations:** ^1^Robotics, Brain and Cognitive Science, Istituto Italiano di TecnologiaGenova, Italy; ^2^iCub Facility, Istituto Italiano di TecnologiaGenova, Italy

**Keywords:** visual attention, neuromorphic, humanoid robotics, event-driven, saliency map

## Abstract

Fast reaction to sudden and potentially interesting stimuli is a crucial feature for safe and reliable interaction with the environment. Here we present a biologically inspired attention system developed for the humanoid robot iCub. It is based on input from unconventional event-driven vision sensors and an efficient computational method. The resulting system shows low-latency and fast determination of the location of the focus of attention. The performance is benchmarked against an instance of the state of the art in robotics artificial attention system used in robotics. Results show that the proposed system is two orders of magnitude faster that the benchmark in selecting a new stimulus to attend.

## 1. Introduction

For successfully interacting with the environment in daily tasks, it is crucial to quickly react to ubiquitous dynamic stimuli. However, reaction time of state-of-the-art robotic platforms is limited by the low temporal resolution of sensory data acquisition and by the time required to process the corresponding sensory data.

In conventional robotic systems, sensory information is available in a sequence of “snapshots” taken at regular intervals. Highly redundant data are received at fixed frame-rate. High dynamics can be sensed only by increasing the sampling rate, at the cost of increasing the quantity of data that needs to be transmitted, stored and processed.

Additionally, the available bandwidth limits the amount of information that can be transmitted, and the available computing platforms limit the speed at which data can be processed, forcing a compromise between resolution and speed. As a result, current robotic systems are less efficient in reacting appropriately to unexpected, dynamic events (Delbruck, 2008). For example, in robotic soccer competitions (e.g., Robocup, 2011), the performance strongly depends on the latencies in the perception loop, where the robot has to detect, track and predict the trajectory of the ball, to plan where and when it should be catched. For the same reason, in manipulation tasks, unexpected failures of the grasping are difficult to correct online, resulting in the fall of the object to be grasped. On the contrary, robotic systems equipped with vision neuromorphic chips show remarkable performance in tracking (Serrano-Gotarredona et al., 2009), ball goalkeeping and pencil balancing (Conradt et al., 2009).

Differently from main-stream state-of-the art vision systems that repeatedly sample the visual input, event-driven vision (Camunas-Mesa et al., 2012; Wiesmann et al., 2012) samples changes in the visual input, being driven by the stimuli, rather than by an external clock. As such, event-driven systems are inherently more efficient because they acquire, transmit and perform computation only when and where a change in the input has been detected, removing redundancies at the lowest possible level. Selective attention is a key component of artificial sensory systems; in robotics, it is the basis for object segmentation (Qiaorong et al., 2009), recognition (Miau et al., 2001; Walther et al., 2005) and tracking (Ouerhani et al., 2005), for scene understanding and action selection for visual tracking and object manipulation. It is also used in navigation, for self-localization and simultaneous localization and mapping (SLAM) (Frintrop and Jensfelt, 2008). Moreover, the implementation of biologically inspired models of attention is helpful in robots that interact with human beings. Engaging attention on similar objects can be the basis for a common understanding of the environment, of shared goals and hence of successful cooperation. State-of-the art artificial attention systems, based on traditional video acquisition, suffer from the high computational load needed to process each frame. Extreme computational demand limits the speed of the selection of new salient stimuli and therefore the dynamics of the attention scan path. Specific implementations of such models have been explicitly developed for real-time applications, exploiting either parallelization on several CPUs (Itti, 2002; Siagian et al., 2011) or dedicated hardware (Ouerhani and Hügli, 2003), or the optimization and simplification of the algorithms (Itti et al., 1998; Frintrop et al., 2007) for the extraction of features from images, or combination of them (Bumhwi et al., 2011).

An alternative approach is the implementation of simplified models of attention systems based on frame-less event-driven neuromorphic vision sensors, so far realized with the design of *ad hoc* dedicated hardware devices (Bartolozzi and Indiveri, 2009; Sonnleithner and Indiveri, 2012).

Along this line of research, we developed an event-driven, attention system capable of selecting interesting regions of the visual input with a very short latency. The proposed system exploits low latency, high temporal resolution and data compression given by event-driven dynamic vision sensors, as well as an efficient strategy for the computation of the attention model that directly uses the output spikes from the sensors. The proposed implementation is therefore fully “event-driven”, exploiting the advantages offered by neuromorphic sensors at its maximum. Intermediate hybrid approaches can be implemented by reconstructing frames from the events and applying the vast collection of available standard machine vision algorithms. However, this approach would suffer from errors in the frame reconstruction due to drifts in the gray level calculation, it would increase the latency of the response and loose the temporal resolution gained by the use of event-driven sensors, hindering the full exploitation of the neuromorphic approach advantages.

The output of the *Event-Driven Visual Attention (EVA)* system has been implemented for the humanoid robot iCub which will therefore be able to quickly orient its gaze, scrutinize and act on the selected region and react to unexpected, dynamical events. Additionally, it can be of generic interest for robotics systems with fast actuation.

In the following, we will describe EVA, show the improved latency in the selection of salient stimulus and compare its performance with the well-known state-of-the art frame-based selective attention system from the iLab Neuromorphic Vision C++ Toolkit (iNVT),^1^ developed at the University of Southern California.

## 2. Methods

The selective attention system described in this work has been developed on the iCub humanoid robot (www.icub.org) and is entirely based on the input from non-standard sensors. Such sensors use a new way of encoding information based on a custom asynchronous communication protocol Address Event Representation (AER). In the following we shortly describe the custom hardware and software modules developed for the attention system implementation.

### 2.1. Hardware

The robot is equipped with two asynchronous bio-inspired *Dynamic Vision Sensors (DVS)* (Lichtsteiner et al., 2008). It features three degrees of freedom in the eyes to realize the tilt, vergence and version movements required for the implementation of active vision. As opposed to the traditional “frame-based” approach, in the DVS each pixel responds to local variations of contrast. It emits an asynchronous digital pulse (“spike” or “event”) when the change of the logarithm of light intensity exceeds a pre-defined threshold. This bio-inspired sensory transduction method is inherently efficient, as it discards redundancies at the lowest level, reducing the data acquisition, transfer, storage and processing needs. This technique preserves the high dynamic content of the visual scene with a temporal granularity of few hundreds of nanoseconds.

The visual system is entirely based on the AER protocol (Deiss et al., 1998). The sensors asynchronously send digital spikes or “events” that signal a relative contrast change in the pixel. The address transmitted with the event corresponds to the identity of the active pixel. Information is self encoded in the timings of the spikes.

A dedicated printed circuit board located in the head of the robot hosts a Field Programmable Gate Array (FPGA) and an embedded processor specialized for asynchronous data, the General Address Event Processor (GAEP) (Hofstaetter et al., 2010). The FPGA merges the data streams from left and right camera sensors and interfaces them with the GAEP. The GAEP provides effective data processing, protocol verification and accurate time-stamping of the events, with a temporal resolution of **160 ns**. Processed events are connected to the rest of the system thanks to an USB connection to a PC104 embedded CPU. The PC104 gathers the data and passes them to the processing infrastructure of the iCub (Metta et al., 2006).

### 2.2. Software

An application running on the embedded PC104 configures the sensors in the preferred operating state. The same software module reads the data through the USB port, checking for protocol errors and formatting the stream of asynchronous events. Each address event (AE) is composed of the emitting pixel address and the corresponding time-stamp. The application sends the received collection of events on the gigabit network where distributed processing takes advantage from middleware YARP^2^ library. From this point, any process connected to the network can acquire data and perform computation. There is no limit in the number of nodes that can be recruited for processing events.

Finally, specific classes are used to efficiently transmit and (un)mask the AER stream into a dedicated format. The AE format consists in: *address event, polarity, timestamp* and *type*. The structure transparently manages events from the DVS sensor, as well as generic events such as complex objects deriving from clustering and feature extraction (Wiesmann et al., 2012).

Buffers of asynchronous data are handled with a two-threads method. N-buffering is used to guarantee concurrent access to data in process, thus avoiding conflicts and allowing each module to run at the desired rate irrespective of the incoming flow of event. Examples of developed modules are used to: display DVS activity, generate feature maps, perform weighted combination of multiple feature maps.

### 2.3. Event driven visual attention—EVA

**Figure 1:**
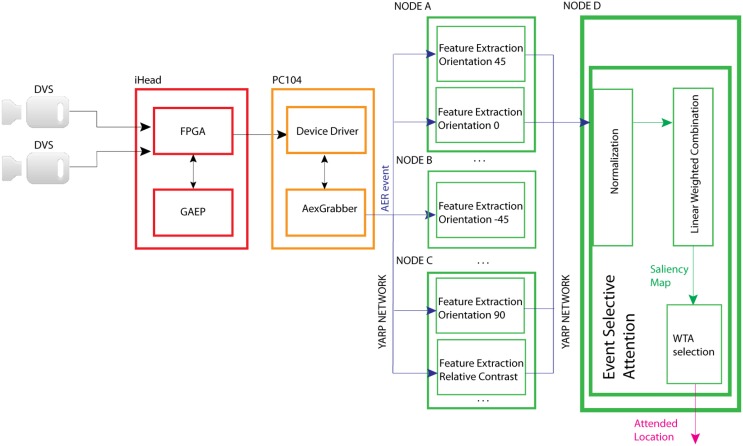
**Structure of EVA and its implementation on the diverse HW modules: the DVS cameras send asynchronous events, the FPGA merges left and right DVS events, the GAEP assigns a timestamp to each event**. The resulting list of addresses and timestamps is sent to the PC104 that makes them available to the YARP network through the AexGrabber module. From then on, any SW module can acquire the events buffer and perform a specific computation (feature extraction, normalization, linear weighted sum, and WTA selection). A dedicated connection between the feature extracting modules and the Event-Selective-Attention module avoids interferences between feature maps and other trains of events.

EVA is an event-driven reduced implementation of the saliency-map based attention model proposed by Koch and Ullman (1985) and Itti and Koch (2001). In this foundational work, the authors propose a biophysically plausible model of bottom–up attention where multiple feature maps concur to form a unique saliency map used to compute the location of the focus of attention. Each feature map encodes for a characteristic of the visual input such as color opponency, orientation, contrast, flicker, motion, etc. computed at different spatial scales. These maps are then normalized and summed together to form the final saliency map. The saliency map topologically encodes for local scene conspicuity, irrespective of the feature dimension that has contributed to its salience. That is, an active location in the saliency map encodes the fact that this location is salient, no matter whether it corresponds to a 45° oriented object in a field of prevalent orientation of 90°, or to a stimulus moving in a static background. Eventually, a winner-take-all (WTA) network selects regions in the map in order of decreasing saliency, and guides the deployment of the focus of attention and gaze. In EVA, events from the visual sensor concur to generate a number of feature maps. Figure 1 shows the model and the distribution of the diverse computing modules on the hardware platform described in paragraph 2.1. Once collected by the dedicated hardware, the sensor's events are sent to software modules that extract diverse visual features. The corresponding feature maps are then normalized and summed. The resulting saliency map is then transmitted to a WTA network that generates the attentional shifts.

#### 2.3.1. Feature extraction

**Figure 2:**
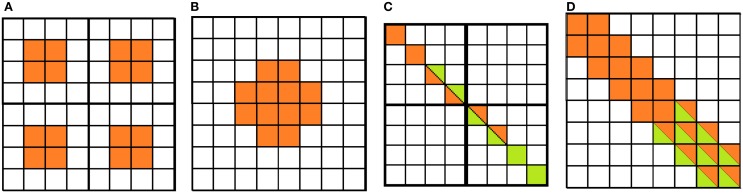
**Receptive fields of cells used for the *mapping* procedure: center-surround cells. (A)** Four 4 × 4 cells, **(B)** one 8 × 8 cell. Simple oriented cells: **(C)** two 4 × 4 cells, **(D)** one 8 × 8 cell. ON- and OFF-regions in orange and white, respectively. In green, the pixels that contribute to the activity of neighboring cells.

In EVA a number of features are extracted from the DVS output to populate diverse feature maps. As the DVS does not convey information about color or absolute intensity, we implemented a subset of feature maps from Itti and Koch (2001): contrast, orientation (0°, 45°, 90°, −45°) and flicker map. Specifically, the flicker map encodes for the scene temporal changes and in EVA it is implemented by directly using the sensor's output. Contrast and orientation feature maps are generated by the output of filters inspired by receptive fields of center-surround retinal ganglion cells and simple cells of primary visual cortex (Hubel and Wiesel, 1962; Movshon et al., 1978; De Valois et al., 1982; Kandel et al., 2000), respectively. Receptive field activation is usually obtained by convolving the image with DOG (Difference of Gaussians) and Gabor filters, respectively. On the contrary, EVA uses a much simpler and efficient implementation: the *mapping*. In the mapping, a RF is defined as a look-up table. The level of activation of the RF increases when it receives ON-spikes from the DVS pixel located in the ON-region of the RF and OFF-spikes in the OFF-region. If the neuron does not receive any spike over time, the activation decreases. When the neuron activation crosses a threshold, it generates a new event in the corresponding location of the feature map. Figures 2A,B show two center-surround RFs. Each RF has a defined location in the visual space and a specific size. The algorithms below explain the procedure that generates the response of the RF.

The visual field is covered with RFs following a multiscale approach. In the current implementation, we use two different scales with 4 × 4 and 8 × 8 pixels receptive fields. Figures 2C,D show the RFs of oriented cells with different sizes. Sub-regions contribute to facilitation for aligned RFs: spikes from the visual field contributing to the activation of the RF at the border of the elongated central region (in green) contribute to the activation of neighboring RFs aligned along the same orientation. This feature enhances the representation of long oriented edges (Ferster and Koch, 1987) by reinforcing the activity of RFs responding to the same oriented edge.

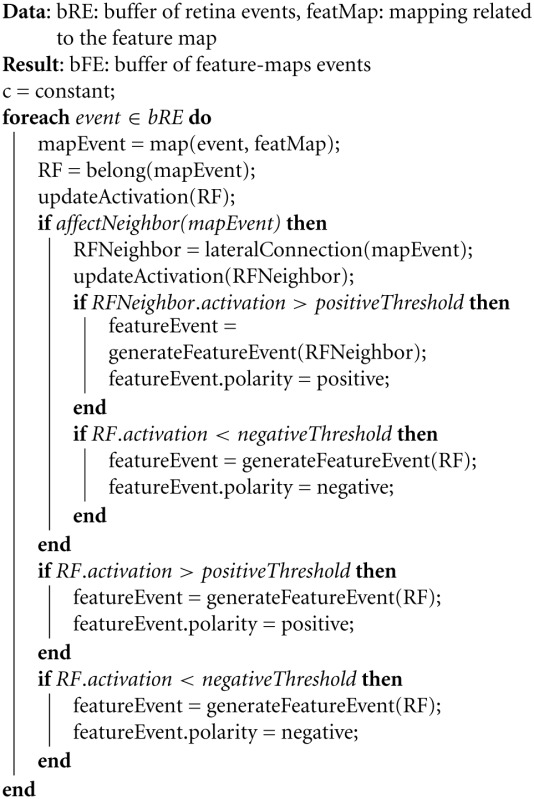

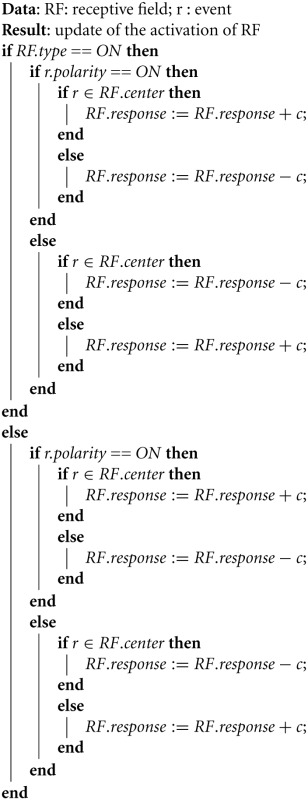





The *mapping* is less computationally demanding than a traditional convolution operation. Additionally, with this approach, the feature maps are only updated at the arrival of a new spike, without calculation of the RF activation for the complete image at each time step. To further reduce the computational load, we implemented non-overlapping receptive fields, at the cost of reducing the output resolution. However, in EVA the final goal is to obtain short latency in relation to saliency map resolution that guarantees reliable gaze shift. As a result the selected region is focused in the sensor's fovea for detailed inspection.

#### 2.3.2. Saliency map and attention selection

The final saliency map is obtained through weighted linear combination of the computed contrast (I), orientation (O) feature maps and flicker feature map (*F*):

(1) $$\begin{array}{cc} S=\text{Norm}\left(k_{I}\cdot I+k_{O}\cdot O+k_{F}\cdot F\right) & \left(1\right) \end{array}$$

The weights *k*_*I*_, *k*_*O*_, and *k*_*F*_ can be changed in real-time to bias saliency computation toward behaviorally relevant features, implementing a task-dependent bias (Itti and Koch, 2001). Finally, a WTA module selects the most conspicuous location of the saliency map, defining the current focus of attention. Feature extraction can be performed in parallel by multiple modules, however, the normalization and sum of feature maps into the saliency map is sequential and requires time. The data-driven system further improves the speed of computation, as the saliency map is updated only with the last train of events, avoiding a complete generation of the entire map.

In iNVT, as well as in most of saliency map based selective attention models, the currently selected location is deselected thanks to a self-inhibition mechanism, known as Inhibition of Return (IOR). This mechanism prevents the system from immediately re-select the current winner, and allows for a scan of many points of the saliency map in order of decreasing conspicuity. However, in our setup neither EVA nor iNVT implement IOR, rather, the shifts of the focus of attention are determined by intrinsic noise in the system.

#### 2.3.3. Ocular movements

A dedicated module implements saccades or gaze shifts toward salient regions selected by EVA. Tremor and microsaccades are used to generate motion of static visual stimuli on the DVS sensor focal plane, to elicit activity of the pixels that only respond to stimulus changes. This approach is similar to the mammals visual system, where small eye movements counteract photoreceptors bleaching adaptation (Kowler, 2011). Tremor is implemented as an omnidirectional movement of 0.45° amplitude with frequency of 500 Hz and random direction, superimposed on microsaccades of amplitude 0.75° and frequency 2.5 Hz in exclusively horizontal direction.

## 3. Performance and benchmark

The absolute novelty of EVA is in the short latency of the attentional shifts that guarantees fast reaction times. The proposed processing of the attention system generates short latency that hardly compares with the performance of frame-based attention systems. The selected attended location can be communicated to the oculomotor controllers to direct the robot's gaze toward salient regions with a saccade command. It continuously updates the saliency map and the resulting focus of attention location, allowing for fast reaction to unexpected, dynamic events and for a more natural interaction of robots with the environment.

The improvement in computation latency is obtained thanks to many factors, among which the asynchronous low-latency and low-redundancy input, efficient sensory encoding, and efficient computing strategy (the mapping).

To assess its performances and validate our results, we tested EVA in three different experimental setups. Unfortunately, a direct quantitative comparison of the performance of EVA with literature state-of-the art artificial bottom–up attention systems cannot be performed as each has its own characteristics in terms of feature maps, methods for feature map calculation, hardware, software implementation, and stimuli (Borji and Itti, 2012). For this reason, we rather preferred to benchmark our implementation against the state-of-the art main-stream system based on the Itti and Koch (2001) model: the *iLab Neuromorphic Vision Toolkit (iNVT)* (Itti et al., 1998, 2003; Navalpakkam and Itti, 2005) sharing the same number and type of feature maps, hardware platform and stimuli. The iNVT algorithm is based on traditional frame-based cameras and convolution operation for the calculation of the feature maps.

The two systems are at the two opposite extremes, one is fully event-driven, the other fully frame-based. Other intermediate solutions might be implemented, where the output of the DVS is first translated into frames by integrating spikes over time, then iNVT is used on the resulting sensory output. However, the necessary transition from event-driven to frame-based information coding spoils some of the advantages of event-driven acquisition, such as temporal resolution and low latency and brings additional costs and relevant overhead in the computation. It is worth to further detail at which extent the performance improvement inherits from the use of DVS sensor as compared to the use of event-based algorithm implementation. As shown in the summary table 2, the latency of EVA amounts to 23 us, of which 15us can be attributed to the characteristic latency of the DVS sensor (Lichtsteiner et al., 2008) and the remaining 8 μs as result of the event-based algorithm. On the contrary, in frame-based scenario, the latency is affected by both the acquisition time (for 30 fps acquisition the acquisition time interval is 33 ms) and the frame-based algorithm for the image processing which we measured in 23 ms. The performance of such systems would be in terms of qualitative performance and computational cost in between the two extremes that are analyzed in the following.

The two systems are implemented on the iCub robot using respectively the DVS and the standard robot's Dragonfly cameras. They simultaneously run on two identical machines^3^; both of them distribute the processing over the four available CPU cores. To correctly compare the two systems, we implemented the same type and number of feature maps in both, restricting the numerous feature maps of iNVT to intensity, orientation and flicker^4^. In order to remove any overhead to the computation time, the iNVT program processes a batch of camera images.

The stimulus is placed at a distance *d* in front of the robot and centered in the fovea of both the Dragonfly and DVS cameras, such that it is completely visible and the quantity of received light is comparable for both sensors. The sensors have been configured with typical parameters (see Table 1) and have not been specifically tuned for the experiments, in order to assess the system's performance in typical use cases.

**Table 1:** **Setup parameters of DVS and Dragonfly sensors**.

**Parameter dragonfly**	**Value**	**Bias DVS**	**Value (μA)**
Width	320 (pixel)	cas	0.094
Height	640 (pixel)	injg	0.0182
Shutter	0.913	reqPd	3.0
Gain	0.312	pux	1.4401
White balance A	0.506	diffoff	2.378*e*^−5^
White balance B	0.494	req	0.0287
Sharpness	0.5	refr	1.688*e*^−4^
Hue	0.48	puy	3.0
Gamma	0.4	diffon	0.1143
Saturation	0.271	diff	0.0054
Framerate	30 (fps)	foll	3.576*e*^−6^
		pr	1.431*e*^−6^

For each experiment we report the diffuse scene light measure^5^ since the performance of both sensors and, consequently, of the two attention systems depend on the illumination level.

For all of the validation setups we report the focus of attention's scan path generated by the two systems, giving an immediate qualitative evaluation of the computation time. For a quantitative assessment the benchmark comprises a set of predefined measurements:

- Number of shifts of the focus of attention over time *F*_EVA_ and *F*_iNVT_ and the correspondent time interval between consecutive shifts Δ*t*_EVA_ and Δ*t*_iNVT_
- CPU utilization *U*_EVA_ and *U*_iNVT_^6^
- Data rate *D*_EVA_ and *D*_iNVT_
- Latency time interval *L*_EVA_ and *L*_iNVT_

The time interval between two consecutive shifts in the selective attention is a good measure of the frequency of attentional redeployments. The latency measure gives an estimate of the minimum reaction time to the new stimuli.

We measure the latency in both systems as the time interval from the instant a novel stimulus is presented to a complete processing of the visual input. In EVA, the latency interval comprises the time interval for feature extraction and WTA selection. The former represents the time necessary to generate a new flow of events associated to feature maps from the moment a new stimulus arrives. The latter represents the time interval to process the generated trains of events and determine attentional shift. In both measures, a sequence of events is needed to alter the output of the module. The frequency of redeployment of the focus of attention depends on the time needed to acquire enough visual data and the time required to extract features, compute saliency and perform the winner-take-all selection. On the contrary, for iNVT we present a single frame and we measure the time interval necessary for the system to process the camera image.

CPU utilization and data rate give an accurate measure of the computation demand of both implementations. To obtain an unbiased measure, we normalized by the number of attentional shifts and report the computational load per shift. The benchmark comprises three test experiments. The first uses typical stimuli for visual experiments, such as oriented gratings, and is run under two different illumination conditions. The second shows the performance of the EVA system with a fast unpredictable stimulus such as a chaotic pendulum. The third indicates how performance changes with the increase of the information to process.

### 3.1. First experiment, gratings with different orientations

**Figure 3:**
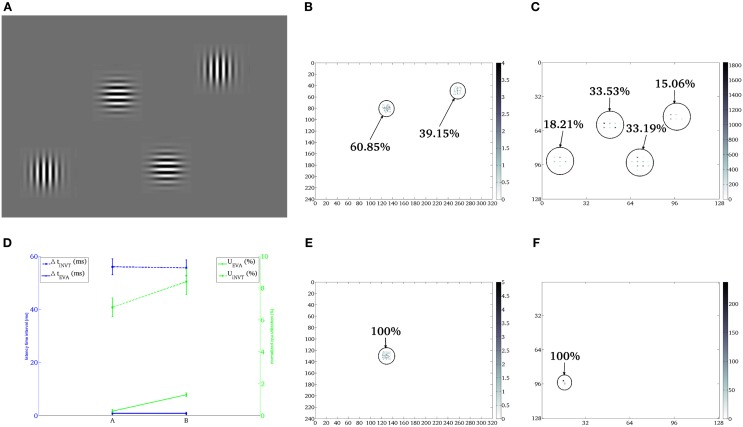
**First scenario**. Case A: comparison of the shifts generated by iNVT **(B)** and EVA **(C)** in response to four oriented gratings **(A)** under bright background illumination (~55.6 LUX—indoor illumination of a diffuse bright natural light). Case B: comparison of the shifts generated by iNVT **(E)** and EVA **(F)** under dim background illumination (~2.7 LUX—dim illumination that typically would require the use of artificial light). The (x,y) coordinates of the two attention systems correspond to the image coordinates of the sensors (240 × 320 for the Dragonfly and 128 × 128 for the DVS). **(D)** Mean and standard deviation of CPU system percentage of utilization (green), temporal distance between consecutive shifts (blue) over 10 repetitions of 10 trials in both illumination conditions.

Figure 3A shows the stimulus used in the first characterization setup: two horizontal and two vertical gratings of 4 × 4 cm with a gaussian profile, each positioned at the distance *d* = 20 cm from the camera. In this scenario the stimuli are static and the DVS output is generated with the use of microsaccades (see section 2.3.3).

#### 3.1.1. Case A, bright illumination

The focus of attention locations selected by EVA and iNVT and their hit frequency are shown in Figures 3C,B, respectively. Both systems select conspicuous locations corresponding to the oriented gratings, with slightly different patterns. As we disabled inhibition of return, the specific focus of attention scan-path depends on the computed saliency and on the noise present in the system. Small differences in stimulus illumination and noise pattern can contribute to slightly different computed saliency for the same grating placed in different regions; the missing inhibition of selected areas over a long period of time leads to the selection of fewer stimuli with very similar salience, as shown in Figure 3B. Two of the oriented gratings are not selected by iNVT, despite they should have had exactly the same salience. In this scenario, EVA is capable of selecting more stimuli, reducing the latency, probably thanks to the different pattern of noise, that is intrinsically generated by the hardware.

In EVA, the data rate depends on the lighting condition and on the stimulus, under these conditions it is about 7 kAE/s. Conversely, the data rate produced by a traditional color camera only depends on intrinsic parameters of the systems such as number of pixels, color resolution and frame rate, being independent from the stimulus; for the Dragonfly used on the iCub this amounts to 530 Mbits/s. The lower amount of data corresponds to lower processing demand and, hence, in a faster computation of the focus of attention location. Consequently this results in higher shifts frequency generated by EVA with respect to iNVT, and higher number of attention relocation, as shown in Table 2. EVA, because of the temporal resolution of the visual signal and the low computational demand, can generate a shift of attention approximately every 1.5 ms, on the contrary, iNVT is limited by the frame acquisition frequency (30 ms) and the time between two attentional shifts amounts to 50 ms. The latencies of the two systems differ of two orders of magnitude, showing the high responsiveness of EVA to sudden and potentially relevant stimuli.

**Table 2:** **Quantitative benchmark: f : frequency of attentional shifts [shifts/s], L : Latency time interval [s], U : normalized cpu utilization [%], D : data rate of input [Kbit/s], δ: duration of the acquisition[s]**.

	**Experiment 1**				**Experiment 2**
	**Bright (~55.6 LUX)**		**Dim (~2.7 LUX)**		**~27.2 LUX**
	**iNVT**	**EVA**	**iNVT**	**EVA**	**EVA**
hor. top	60.85%	33.53%	100%	0%	.
hor. bot	0%	33.19%	0%	0%	.
ver. top	39.15%	15.06%	0%	0%	.
ver. bot	0%	18.21%	0%	100%	.
f (shifts/s)	1.89	158.2	18.08	3.72	1708.80
L (s)	(5.60 ± 0.3)*e*^−2^	(23.2 ± 3)*e*^−4^	(5.56 ± 0.3)*e*^−2^	(23.1 ± 3)*e*^−4^	(3.72 ± 1)*e*^−3^
U (%)	6.79	0.2	8.4	1.3	0.2
D (Kbit/s)	530*e*^3^	226.72 ± 0.078	530*e*^3^	20.32 ± 1.2	2.1*e*^3^
δt (s)	100	100	100	100	2.68

*First experiment: number of hits clustered on the horizontally (top and bottom) and vertically (top and bottom) oriented grating stimuli under bright and dim illumination. Second experiment: performance of the EVA in details*.

Figure 3 shows that the computation demand of EVA is lower than iNVT of at least one order of magnitude, as expected from the lower Data Rate and the different computational load of the mapping procedure. This different performance is also reflected in the shift latency that amounts to about 300 ns for EVA and 0.4 ms for iNVT.

#### 3.1.2. Case B, dim illumination

One of the advantages of using the logarithmic encoding in the DVS is the wider dynamic range with respect to traditional sensors. Thus, we tested the attention systems in the scenario described above, but with reduced ambient light. The resulting focus of attention scan path is shown in Figures 3E,F. The selection of the top horizontally oriented grating in the iNVT system and the selection of the bottom vertical oriented grating in EVA are the results of a strong decrease of the response strength. The lower illumination affects both systems by drastically reducing the number of shifts. A way to improve this behavior in EVA would be the implementation of adaptive firing threshold, that can be dynamically set according to the level of background illumination (Delbruck and Oberhof, 2004).

Figure 3 shows the aggregated performance measures for case A and B for both EVA and iNVT; Despite the latency of both systems remains largely unchanged, EVA outperforms iNVT, while the normalized computational load increases, with different slopes. In case B (low illumination), iNVT absolute CPU usage remains unchanged but it is normalized for a lower number of shifts; in EVA both the number of shifts and the CPU utilization decrease, as a result of a lower input data rate, as shown in Table 2. The resulting normalized computation load increases less than what observed for iNVT.

### 3.2. Second experiment: chaotic pendulum

**Figure 4:**
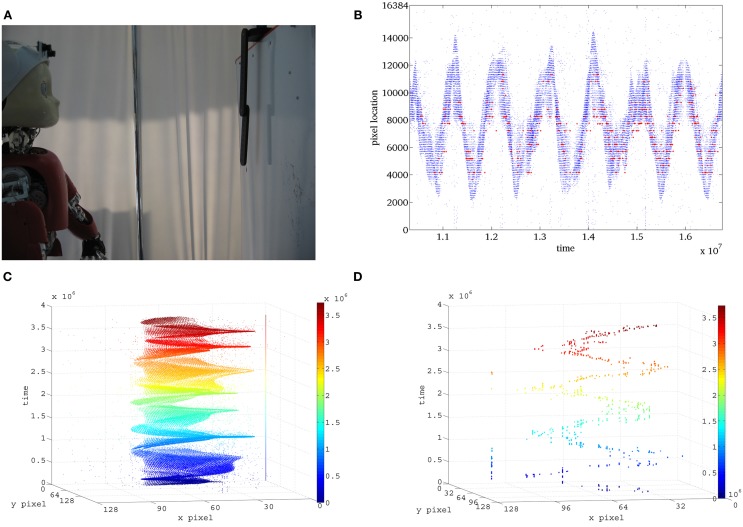
**Second scenario. (A)** The chaotic pendulum is located 50 cm far from the robot to keep the whole stimulus in the camera's field of view. In the setup, DVS sensors are embedded in the iCub's eyes to exploit ocular movements, while the Dragonfly cameras are mounted on the head with fixed supports. **(B)** Raster representation of the activation of pixels in the DVS and relative location of the WTA selected by EVA. **(C)** Events generated by the chaotic pendulum, **(D)** focus of attention scan-path generated by EVA.

We used a chaotic pendulum to test EVA with fast unpredictable stimuli. The chaotic pendulum shown in Figure 4A is composed of two black bars (22 and 18 cm) connected by a low friction joint and attached to a fixed support via a second low friction joint. In this configuration, the first bar can freely rotate with respect to the support and its movement is influenced by the second bar that revolves independently around the first joint. The pendulum is mounted over a white background and we used an average lighting condition of 27.6LUX—corresponding to diffuse illumination where artificial light is not required.

The stimulus is so fast that neither the Dragonfly, nor the human eye, can successfully perceive its full motion. In this scenario, iNVT hardly relocates the focus of interest on the pendulum without introducing an evident delay. In iNVT, such shift is clearly shown in the video provided in Technical Materials.^7^ Conversely, we accurately assess the performance of EVA as a viable to technological solution for fast dynamic stimuli in a wide range of operating conditions.

The fast movement of the pendulum generates a higher data rate with respect to the previous scenario. The resulting performance parameters are listed in Table 2.

To estimate the quality of the attention system, in Figure 4 we compare the trajectory generated by the pendulum with the location of the attention shifts over time. To achieve this, we synchronized the generation of attention shifts with the batch data of the generated events, using the temporal information stored in the timestamp.

### 3.3. Third experiment: performance scaling with quantity of information

**Figure 5:**
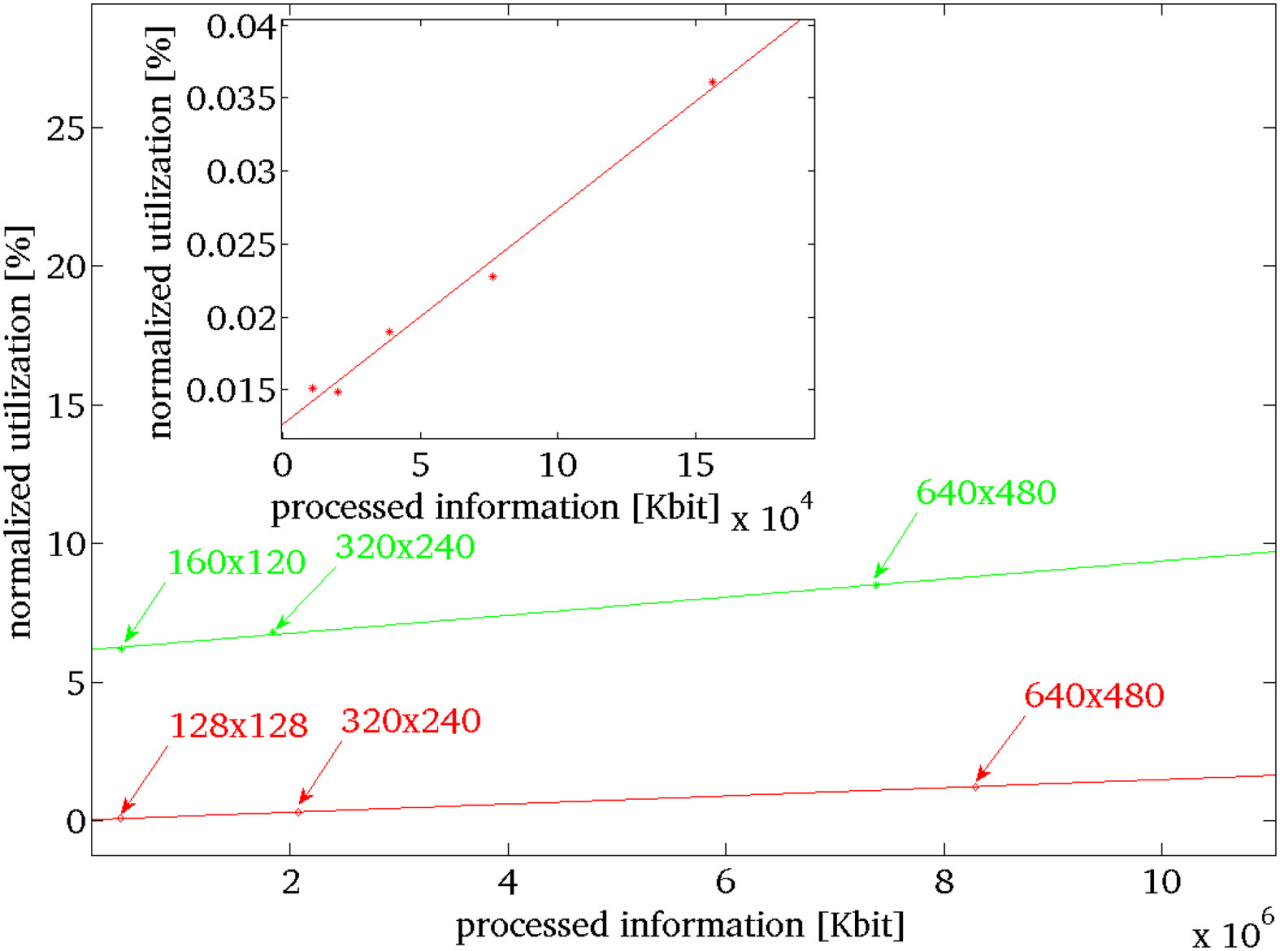
**Expected evolution of the normalized computation demand for increasing the sensor's output by increasing the sensor's size, in green iNVT and in red EVA**. In the inset the measured data for EVA, for increasing number of events, generated by increasing number of black rotating edges repeated, respectively, at 360°, 180°, 90°, 45°, and 22.5°, in red the fit from which we extrapolate the computational demand for bigger sensors.

In this scenario, we assess how the performance of EVA scales with increasing number of events. In EVA, the number of events can increase for cluttered scenes and for higher resolution sensors, increasing the computational demand of the system. This happens also for iNVT, when higher resolution sensors are used. We estimate how the computation demand expressed in CPU utilization scales with the processed information (number of bits). In order to perform such analysis we determined how the computation demand of the two systems change when the quantity of information scales. For EVA, we control the number of generated events (and then quantity of information) by increasing the number of black edges printed on a white disk rotating at constant speed. We use five different configurations, where the edge is repeated every 360°, 180°, 90°, 45°, and 22.5°. Figure 5 shows the normalized CPU utilization measured in this experiment (red dots in the inset); we do a linear fit of the normalized CPU utilization in relation with the increasing quantity of information processed. We use this function as reference to estimate the computation demand of EVA at arbitrary number of generated events. Similarly, for iNVT, we provide different sets of images. The sets differ only for the dimension of the images, the stimulus is the same of Figure 4A. The computation demand increases with the quantity of processed information (green dots in Figure 5) and we fit a first order curve that best describes the distribution.

Figure 5 shows that the required level of processing for EVA (in red) never exceeds the required level of processing of iNVT (in green) as it increases with a more gradual slope. Figure 5 shows that the required level of processing for EVA (in red) never exceeds the required level of processing of iNVT (in green) as it increases with a more gradual slope. This observed divergence indicates the increasingly better performance of EVA, as more processing is required. Key points (arrows in figure) help identifying the estimated computation demand for both the systems in correspondence of quantity of information generated by different fixed size sensors (128 × 128, 320 × 240, 640 × 480).

To estimate these numbers, we select speed of rotating bar that covers typical use (4.2284 rad/s). Even though in normal scene operation the DVS activation is about 30%, in this test, we consider the worst case scenario where all the pixels in the sensor show the maximum level of activation (27 events per pixel).

Thus, we estimate the maximum computation load associated to sensors that have dimension 128 × 128, like the DVS, 320 × 240, like the Dragonfly used for iNVT and 640 × 480. As the processing required by EVA sets well below iNVT, we conclude that for any possible situation, the required processing of EVA results less impacting on the performance than iNVT.

This confirms the assumption that relevant saving in computation demand is associated to the design of the processing in EVA and it is not limited to the hardware of the system.

## 4. Discussion

In this manuscript we described EVA, a real-time implementation of selective attention based on the bio-inspired model proposed in the foundational work of Itti and Koch (2001), that uses a frame-less asynchronous vision sensor as input. The goal of the implementation was to offer a flexible and light computational paradigm for the computation of the feature maps, exploiting the *mapping* mechanism. The overall performance of the developed system takes advantage of the efficient information encoding operated of the sensor, its high dynamic range, low response latency and high temporal resolution. The use of non-conventional sensors coupled with an efficient processing results in a unprecedented fast attentional selection. We report the behavior of the system in three different experiments that highlight performance in detail. The three experiments give insights on the three major benefits of EVA: low computation demand, high responsiveness to high frequency stimuli and favorable scalability. The attention system EVA requires lower computation utilization up to one order of magnitude with respect to iNVT when stimulated by identical stimulus. This positive characteristic does not degrade the quality of the generated attention shifts. The characteristic of low response latency and high temporal resolution resulting from the efficient design of the attention system EVA allow remarkable performance in highly dynamic scenarios. The attention system accurately and swiftly redeploys the attentional foci on the most salient regions in the visual field even in situations where frame-based algorithms of visual attention fail in obtaining clear interpretation of the stimulus. The second scenario shows that the high temporal resolution allows the attention system to track very fast stimuli, expanding the application range of the system from humanoid robotics to even more demanding use cases. We presented a solution that, by sensing via efficient event-driven hardware sensor, provides outperforming selective attention mechanism with low latency and high dynamic range. In addition, for increasing the information load, e.g., for higher resolution sensors, EVA's CPU utilization increases with lower rate than iNVT's. The design of efficient processing in EVA guarantees, when compared with iNVT, relative superior performance of growing effectiveness with the amount of processed information. Finally, the last benchmark shows that the computational advantage of EVA is not restricted to the specific stimuli and sensor dimension used in this experimental setup, rather is more general.

Most attention systems designed for real-time applications report the computational cost in terms of time needed to process a frame. The relative saliency map is often obtained in about 50–60 ms, slower than typical image frame-rate (30 frames per second) (Frintrop et al., 2007). This time scale is appropriate to reproduce typical attentional scan-paths, nevertheless, 50 ms (plus 30 ms for frame acquisition) is the lower bound for reacting to the onset of a new potentially interesting or threatening stimulus. With EVA, this limit is estimated to be as small as about 1 ms [plus 15 μs of sensor latency (Lichtsteiner et al., 2008)], thanks to the low-latency event-driven front-end data acquisition and the low computational cost of the attention system. This property is crucial in robotics systems, as it allows the robot to plan actions for reacting to unforeseen situations and sudden stimuli.

EVA has been developed to equip the iCub with a fast and low weight attention system, exploiting the event-driven vision system of the iCub (Bartolozzi et al., 2011). The mapping procedure for events filtering and feature maps generation derives from AER implementations (Bartolozzi and Indiveri, 2009; Sonnleithner and Indiveri, 2012), where a simple mapping is realized to use the sensor output as feature map. The resulting saliency map is sent to a dedicated hardware platform that implements the winner-takes-all selection enriched with dedicated inhibition of return mechanism (the Selective Attention Chip, SAC). The modules developed in this work and EVA can easily be integrated with such a system and further optimized. For example, maximization of the performance can be achieved by implementing the mapping procedure and the relative feature maps on an embedded FPGA (Bartolozzi et al., 2011; Fasnacht and Indiveri, 2011) or implementing fast convolution on ConvNet chips (Serrano-Gotarredona et al., 2008) and using the SAC (or higher resolution implementations) for WTA and IOR. Both implementations would probably be faster than the software mapping procedure described in this manuscript, for example, the ConvNet chip can start providing the output of oriented filters with a 1 μs latency and is shown to perform pseudo-simultaneous object recognition. This system, with the appropriate miniaturization and integration with top–down modules implemented on the robot, will be able to give a fast estimate of the focus of attention, leaving the computational units of the iCub free for other more complex tasks.

### Conflict of interest statement

The authors declare that the research was conducted in the absence of any commercial or financial relationships that could be construed as a potential conflict of interest.

## Footnotes

^1^http://ilab.usc.edu/toolkit/home.shtml

^2^Yet Another Robotic platform.

^3^Intel Core 2 Quad Cpu Q9950 @2.83GHz

^4^*ezvision −in=raster:^*^.ppm −display=display -T -j 4 −input-frames=@30Hz −textlog=iNVTLog.log −vc-chans=IOF −ior-type=None −use-random.*

^5^Measured by portable hand-held exposure meter Gossen Lunasix F.

^6^Measurements performed with *SAR*, a program that directly measures the computational load on the processor over a user-defined time interval.

^7^http://youtu.be/Nqd3uRbjXHE
